# Response to Dr. Randolph and Drs. Gern and Humair

**DOI:** 10.3201/eid0606.000622

**Published:** 2000

**Authors:** Dania Richter, Andrew Spielman, Nicholas Komar, Franz-Rainer Matuschka

**Affiliations:** * Charité, Medizinische Fakultät der Humboldt-Universität zu Berlin, Berlin, Germany;; † Harvard School of Public Health, Boston, Massachusetts, USA;; ‡ Current affiliation: Centers for Disease Control, Fort Collins, Colorado, USA


repeated infestations of ticks on mice, even without obvious reduced feeding success, result in reduced transmission of spirochetes between mice and ticks (19).

**Sarah Randolph**
University of Oxford, Oxford, United Kingdom

### References

1. Richter D, Spielman A, Komar N, Matuschka F-R. Competence of American robins as reservoirs for Lyme disease spirochetes. Emerg Infect Dis 2000;6:133-8.
2. Slajchert TL, Kitron UD, Jones CJ, Mannelli A. Role of the eastern chipmunk (*Tamias striatus*) in the epizootiology of Lyme borreliosis in northwestern Illinois, USA. J Wild Dis 1997;33:40-6.
3. Craine NG, Nuttall PA, Marriott AC, Randolph SE. Role of grey squirrels and pheasants in the transmission of *Borrelia burgdorferi* sensu lato, the Lyme disease spirochaete, in the U.K. Folia Parasitol 1997;44:155-60.
4. Humair P-F, Gern L. Relationship between *Borrelia burgdorferi* sensu lato species, red squirrels *(Sciurus vulgaris*) and *Ixodes ricinus* in enzootic areas in Switzerland. Acta Trop 1998;69:213-27.
5. Gern L, Rouvinez E, Toutoungi LN, Godfroid E. Transmission cycles of *Borrelia burgdorferi* sensu lato involving *Ixodes ricinus* and/or *I. hexagonus* ticks and the European hedgehog, *Erinaceus europaeus*, in suburban and urban areas in Switzerland. Folia Parasitol 1997;44:309-14.
6. Ogden NH, Randolph SE, Nuttall PA. Natural Lyme disease cycles maintained via sheep by co-feeding ticks. Parasitology 1997;115:591-9.
7. Humair P-F, Postic D, Wallich R, Gern L. An avian reservoir (*Turdus merula*) of the Lyme borreliosis spirochete. Zentralbl Bakteriol 1998;287:521-38.
8. Matuschka F-R, Spielman A. Loss of Lyme disease spirochetes from *Ixodes ricinus* ticks feeding on European blackbirds. Exp Parasitol 1992;74:151-8.
9. Kurtenbach K, Carey D, Hoodless AN, Nuttall PA, Randolph SE. Competence of pheasants as reservoirs for Lyme disease spirochetes. J Med Entomol 1998;35:77-81.
10. Randolph SE, Craine NG. General framework for comparative quantitative studies on transmission of tick-borne diseases using Lyme borreliosis in Europe as an example. J Med Entomol 1995;32:765-77.
11. Kurtenbach K, Peacey MF, Rijpkema SGT, Hoodless AN, Nuttall PA, Randolph SE. Differential transmission of the genospecies of *Borrelia burgdorferi* sensu lato by game birds and small rodents in England. Appl Environ Microbiol 1998;64:1169-74.
12. Humair P-F, Peter O, Wallich R, Gern L. Strain variation of Lyme disease spirochetes isolated from *Ixodes ricinus* ticks and rodents collected in two endemic areas in Switzerland. J Med Entomol 1995;32:433-8.
13. Hu CM, Humair P-F, Wallich R, Gern L. *Apodemus* sp. rodents, reservoir hosts for *Borrelia afzelii* in an endemic area in Switzerland. Zentralbl Bakteriol 1997;285:558-64.
14. Kurtenbach K, Sewell H, Ogden NH, Randolph SE, Nuttall PA. Serum complement sensitivity as a key factor in Lyme disease ecology. Infect Immun 1998;66:1248-51.
15. Humair P-F, Rais O, Gern L. Transmission of *Borrelia afzelii* from Apodemus mice and *Clethrionomys* voles to *Ixodes ricinus* ticks: differential transmission pattern and overwintering maintenance. Parasitology 1999;118:33-42.
16. Gigon F. Biologie d'*Ixodes ricinus* L. sur le Plateau Suisse—une contribution à l'écologie de ce vecteur [thesis]. Neuchätel, Switzerland: l'Université de Neuchätel: 1985.
17. Randolph SE, Storey K. Impact of microclimate on immature tick-rodent interactions (Acari: Ixodidae): implications for parasite transmission. J Med Entomol 1999;36:741-8.
18. Rogers DJ. A general model for the African trypanosomiases. Parasitology 1988;97:193-212.
19. Wikel SK, Ramachandra RN, Bergman DK, Burkot TR, Piesman J. Infestation with pathogen-free nymphs of the tick *Ixodes scapularis* induces host resistance to transmission of *Borrelia burgdorferi* by ticks. Infect Immun 1997;65:335-8.

### Response to Dr. Randolph and Drs. Gern and Humair

**To the Editor:** We define reservoir competence of a host for a vector-borne pathogen in terms of three component questions: How susceptible is the putative reservoir host when the pathogen is delivered by the bite of an infected vector tick? How effectively does the pathogen proliferate and develop in this host? And how infective is the resulting infected host to vector ticks and for how long (1,2)? Drs. Gern and Humair insert the parenthesis (implied xenodiagnosis) into a citation of our text, thereby, equating reservoir competence with a simple xenodiagnostic test that partially addresses only the third component of this definition. At best, such a test records degree of infectivity to vector ticks at some arbitrary and often unknown point in time, a consideration that persuades us to limit our citations referring to reservoir competence. Conclusions derived from xenodiagnosis performed on field-derived animals differ from those that are obtained by an experimental study. With regard to acknowledging relevant research, we did cite the study on pheasants (3) in which these birds were infected in the laboratory by tick-borne spirochetes and subsequently infected only about a quarter of vector ticks. The cited study on blackbirds (4), on the other hand, used ticks solely to diagnose infection in field-derived birds that had been infected in nature. Although a few of these animals proved to be infectious to xenodiagnostic ticks when tested 1-3 days after capture, this study failed to quantify susceptibility or to determine intensity and duration of infectiousness to vector ticks. Our rigorously standardized study (1) is the first to establish experimentally that birds are highly competent as reservoir hosts for Lyme disease spirochetes.

Drs. Gern and Humair disagree with our statement that "larval ticks seem not to feed on [pheasants], either in the laboratory or in nature." However, a field study on pheasants states that "no fully engorged larvae ... were recovered from thirty adult male pheasants shot in a Dorset woodland" (5). The previously cited experimental study on these birds similarly demonstrated that larval infestations generally fail, and stated that "In fact, most of the introduced larvae died while attempting to feed on the pheasants" (3). Inflammatory responses directed against feeding larvae were advanced as a possible explanation for the observed failure to feed to repletion. The larval stage of the American vector similarly seems to feed poorly on chickens (6). Passerine birds, however, seem to serve effectively as hosts for the larval stages of this complex of ticks, and we find that larvae attach readily to American robins (1). Numerous larval *Ixodes ricinus* ticks feed on European blackbirds in nature, and larval ticks attach readily and repeatedly to such birds in the laboratory (7,8). Therefore, the limited attractiveness of gallinaceous birds to larval *Ixodes* ticks may render them less important than certain passerine birds as natural reservoir hosts for Lyme disease spirochetes.

The transmission cycle of the agent of Lyme disease tends to be more complex in Europe than in North America. The host range of the European vector tick, *I. ricinus*, is broader than that of its American cousin (*I. dammini*, frequently cited as *I. scapularis*), and the European pathogen in humans is more diverse, comprising several genospecies. Our study aimed to define the competence of the American robin, *Turdus migratorius*, as a reservoir host for rodent-infecting *Borrelia burgdorferi* sensu stricto—not *B. burgdorferi* sensu lato as stated in Drs. Gern and Humair's letter—and used American *Ixodes* ticks as vectors. The mode of perpetuation of the agents of human Lyme disease in Europe is peripheral to the subject of our article.

Dr. Randolph and Drs. Gern and Humair express commitment to the concept that different European spirochetal genospecies perpetuate simultaneously in distinct kinds of vertebrate reservoir hosts. Their concept requires that a larval *I. ricinus* tick that acquires a rodent-specific genospecies from a rodent host must, in its nymphal stage, again feed on a rodent. If this nymphal tick were to feed on a bird, the rodent-specific spirochete would not perpetuate because this nonpermissive host would function zooprophylactically. A suggested avian-specific spirochete would perpetuate reciprocally. According to the MacDonald concept of vectorial capacity (9), such a relationship would be unlikely if pathogens requiring different reservoir host populations were to be transmitted simultaneously by the same vector population. The studies cited in support of this concept rest on correlative evidence derived from field data. No confirmatory experimental proof demonstrates an especially intense association of *B. afzelii* with rodents and *B. garinii* or *B. valaisiana* with birds. Indeed, European larval ticks acquire *B. afzelii* as well as *B. garinii* infection from field-derived passerine birds (10). Various other observations also contradict the suggested close association between genospecies and particular kinds of hosts (11-13). One of the studies (14) cited as evidence for genospecies specificity was published even before the genospecies were differentiated; the other "consistent independent findings" derive from the laboratories of Drs. Randolph and Gern. Our findings that birds serve as competent hosts for an apparently mammal-perpetuated spirochetal genospecies would seem to contradict the concept of separate genospecies perpetuation. No rigorous evidence is yet in hand to support the theory that the same population of vector ticks perpetuates different European spirochetal genospecies differentially in particular kinds of reservoir hosts.

Dr. Randolph suggests that our experiments may have been confounded because Lyme disease spirochetes may have been inherited persistently within the laboratory colony of ticks used in our studies. Although an early observation points toward the possibility of such a mode of transovarial transmission

(15), subsequent experimental evidence suggests that vertical transmission rarely, if ever, occurs (16). Inherited infection in nature would be exceedingly infrequent because spirochetes infect less than 1% of naturally questing larvae, both in North America and in Europe (17, 18), and some of these larvae may have acquired infection by feeding partially on an infected host. We routinely seek evidence of spirochetal infection in each cohort of larval ticks used in our experiments but have never found spirochetes in a nonfed, laboratory-reared larva. Our reported frequency of experimental transmission of Lyme disease spirochetes from reservoir mice to vector ticks corresponds to that reported elsewhere (19-21).

Dr. Randolph's statistical analysis of our data confirms that the feeding success of nymphal ticks on robins exposed repeatedly to ticks varies nonsignificantly, supporting our conclusion that nymphal ticks readily feed repeatedly on tick-exposed robins. Although repeated nymphal infestations may protect inbred laboratory mice from tick-borne spirochetes (22), natural reservoir hosts, such as white-footed mice and American robins, remain susceptible to such spirochetes, regardless of prior exposure to ticks (1, 23).

**Dania Richter,*† Andrew Spielman,† Nicholas Komar,†‡ and Franz-Rainer Matuschka*†**

*Charité, Medizinische Fakultät der Humboldt-Universität zu Berlin, Berlin, Germany; †Harvard School of Public Health, Boston, Massachusetts, USA; ‡Current affiliation: Centers for Disease Control, Fort Collins, Colorado, USA.

### References

1. Richter D, Spielman A, Komar N, Matuschka F-R. Competence of American robins as reservoir hosts for Lyme disease spirochetes. Emerg Infect Dis 2000;6:133-8.
2. Spielman A, Rossignol PA. Principles of public health entomology. In: Warren KS, Mahmoud AAF, editor. Tropical and geographical medicine. New York: McGraw-Hill; 1984. p. 167-83.
3. Kurtenbach K, Carey D, Hoodless AN, Nutall PA, Randolph SE. Competence of pheasants as reservoirs for Lyme disease spirochetes. J Med Entomol 1998;35:77-81.
4. Humair P-F, Postic D, Wallich R, Gern L. An avian reservoir (*Turdus merula*) of the Lyme borreliosis spirochetes. Zentralbl Bakteriol 1998;287:521-38.
5. Kurtenbach K, Peacey M, Rijpkema SGT, Hoodless AN, Nutall PA, Randolph SE. Differential transmission of the genospecies of *Borrelia burgdorferi* sensu lato by game birds and small rodents in England. Appl Environ Microbiol 1998;64:1169-74.
6. Piesman J, Dolan MC, Schriefer ME, Burkot TR. Ability of experimentally infected chickens to infect ticks with the Lyme disease spirochete, *Borrelia burgdorferi*. Am J Trop Med Hyg 1996;54:294-8.
7. Walter G. Vorkommen und Biologie von Vogelzecken (Ixodidae) in Deutschland – Eine Übersicht. Abhandlungen aus dem Gebiet der Vogelkunde 1979;6:163-70.
8. Matuschka F-R, Spielman A. Loss of Lyme disease spirochetes from *Ixodes ricinus* ticks feeding on European blackbirds. Exp Parasitol 1992;74:151-8.
9. MacDonald G. The epidemiology and control of malaria. London: Oxford University Press; 1957.
10. Olsen B, Jaenson TGT, Bergström S. Prevalence of *Borrelia burgdorferi* sensu lato-infected ticks on migrating birds. Appl Environ Microbiol 1995;61:3082-7.
11. Ishiguro F, Takada N, Nakata K. Reservoir competence of the vole, *Clethrionomys rufocanus bedfordiae*, for *Borrelia garinii* or *Borrelia afzelii*. Microbiol Immunol 1996;40:67-9.
12. Gray JS, Schönberg A, Postic D, Belfaiza J, Saint-Girons I. First isolation and characterization of *Borrelia garinii*, agent of Lyme borreliosis, from Irish ticks. Ir J Med Sci 1996;165:24-6.
13. Richter D, Endepols S, Ohlenbusch A, Eiffert H, Spielman A, Matuschka F-R. Genospecies diversity of Lyme disease spirochetes in rodent reservoirs. Emerg Infect Dis 1999;5:291-6.
14. Hovmark A, Jaenson TGT, Asbrink E, Forsman A, Jansson E. First isolations of *Borrelia burgdorferi* from rodents collected in northern Europe. Acta Pathol Microbiol Immunol Scand Sect B 1988;96:917-20.
15. Burgdorfer W, Barbour AG, Hayes SF, Peter O, Aeschlimann A. Erythema chronicum migrans—a tick-borne spirochetosis. Acta Tropica 1983;40:79-83.
16. Matuschka F-R, Schinkel TW, Klug B, Spielman A, Richter D. Failure of *Ixodes* ticks to inherit *Borrelia afzelii* infection. Appl Environ Microbiol 1998;64:3089-91.
17. Kurtenbach K, Kampen H, Dizij A, Arndt S, Seitz HM, Schaible UE, Simon MM. Infestation of rodents with larval *Ixodes ricinus* (Acari: Ixodidae) is an important factor in the transmission cycle of *Borrelia burgdorferi* s.l. in German woodlands. J Med Entomol 1995:32:807-817.
18. Mejlon HA, Jaenson TGT. Seasonal prevalence of *Borrelia burgdorferi* in *Ixodes ricinus* in different vegetation types in Sweden. Scand J Infect Dis 1993;25:449-56.
19. Donahue JG, Piesman J, Spielman A. Reservoir competence of white-footed mice for Lyme disease spirochetes. Am J Trop Med Hyg 1987;36:92-6.
20. Mather TN, Telford SR, Moore SI, Spielman A. *Borrelia burgdorferi* and *Babesia microti*: efficiency of transmission from reservoirs to vector ticks (*Ixodes dammini*). Exp Parasitol 1990;70:55-61.

21. Randolph SE, Craine NG. General framework for comparative quantitative studies on transmission of tick-borne diseases using Lyme borreliosis in Europe as an example. J Med Entomol 1995;32:765-77.
22. Wikel SK, Ramachandra RN, Bergman DK, Burkot TR, Piesman J. Infestation with pathogen-free nymphs of the tick *Ixodes scapularis* induces host resistance to transmission of *Borrelia burgdorferi* by ticks. Infect Immun 1997;65:335-8.
23. Richter D, Spielman A, Matuschka F-R. Effect of prior exposure to noninfected ticks on susceptibility of mice to Lyme disease spirochetes. Appl Environ Microbiol 1998;4596-9.

**To the Editor:** We define reservoir competence of a host for a vector-borne pathogen in terms of three component questions: How susceptible is the putative reservoir host when the pathogen is delivered by the bite of an infected vector tick? How effectively does the pathogen proliferate and develop in this host? And how infective is the resulting infected host to vector ticks and for how long (1,2)? Drs. Gern and Humair insert the parenthesis (implied xenodiagnosis) into a citation of our text, thereby, equating reservoir competence with a simple xenodiagnostic test that partially addresses only the third component of this definition. At best, such a test records degree of infectivity to vector ticks at some arbitrary and often unknown point in time, a consideration that persuades us to limit our citations referring to reservoir competence. Conclusions derived from xenodiagnosis performed on field-derived animals differ from those that are obtained by an experimental study. With regard to acknowledging relevant research, we did cite the study on pheasants (3) in which these birds were infected in the laboratory by tick-borne spirochetes and subsequently infected only about a quarter of vector ticks. The cited study on blackbirds (4), on the other hand, used ticks solely to diagnose infection in field-derived birds that had been infected in nature. Although a few of these animals proved to be infectious to xenodiagnostic ticks when tested 1-3 days after capture, this study failed to quantify susceptibility or to determine intensity and duration of infectiousness to vector ticks. Our rigorously standardized study (1) is the first to establish experimentally that birds are highly competent as reservoir hosts for Lyme disease spirochetes.

Drs. Gern and Humair disagree with our statement that "larval ticks seem not to feed on [pheasants], either in the laboratory or in nature." However, a field study on pheasants states that "no fully engorged larvae … were recovered from thirty adult male pheasants shot in a Dorset woodland" (5). The previously cited experimental study on these birds similarly demonstrated that larval infestations generally fail, and stated that "In fact, most of the introduced larvae died while attempting to feed on the pheasants" (3). Inflammatory responses directed against feeding larvae were advanced as a possible explanation for the observed failure to feed to repletion. The larval stage of the American vector similarly seems to feed poorly on chickens (6). Passerine birds, however, seem to serve effectively as hosts for the larval stages of this complex of ticks, and we find that larvae attach readily to American robins (1). Numerous larval *Ixodes ricinus* ticks feed on European blackbirds in nature, and larval ticks attach readily and repeatedly to such birds in the laboratory (7,8). Therefore, the limited attractiveness of gallinaceous birds to larval *Ixodes* ticks may render them less important than certain passerine birds as natural reservoir hosts for Lyme disease spirochetes.

The transmission cycle of the agent of Lyme disease tends to be more complex in Europe than in North America. The host range of the European vector tick, *I. ricinus*, is broader than that of its American cousin (*I. dammini*, frequently cited as *I. scapularis*), and the European pathogen in humans is more diverse, comprising several genospecies. Our study aimed to define the competence of the American robin, *Turdus migratorius*, as a reservoir host for rodent-infecting *Borrelia burgdorferi* sensu stricto--not *B. burgdorferi* sensu lato as stated in Drs. Gern and Humair's letter--and used American *Ixodes* ticks as vectors. The mode of perpetuation of the agents of human Lyme disease in Europe is peripheral to the subject of our article.

Dr. Randolph and Drs. Gern and Humair express commitment to the concept that different European spirochetal genospecies perpetuate simultaneously in distinct kinds of vertebrate reservoir hosts. Their concept requires that a larval *I. ricinus* tick that acquires a rodent-specific genospecies from a rodent host must, in its nymphal stage, again feed on a rodent. If this nymphal tick were to feed on a bird, the rodent-specific spirochete would not perpetuate because this nonpermissive host would function zooprophylactically. A suggested avian-specific spirochete would perpetuate reciprocally. According to the Macdonald concept of vectorial capacity (9), such a relationship would be unlikely if pathogens requiring different reservoir host populations were to be transmitted simultaneously by the same vector population. The studies cited in support of this concept rest on correlative evidence derived from field data. No confirmatory experimental proof demonstrates an especially intense association of *B. afzelii* with rodents and *B. garinii* or *B. valaisiana* with birds. Indeed, European larval ticks acquire *B. afzelii* as well as *B. garinii* infection from field-derived passerine birds (10). Various other observations also contradict the suggested close association between genospecies and particular kinds of hosts (11-13). One of the studies (14) cited as evidence for genospecies specificity was published even before the genospecies were differentiated; the other "consistent independent findings" derive from the laboratories of Drs. Randolph and Gern. Our findings that birds serve as competent hosts for an apparently mammal-perpetuated spirochetal genospecies would seem to contradict the concept of separate genospecies perpetuation. No rigorous evidence is yet in hand to support the theory that the same population of vector ticks perpetuates different European spirochetal genospecies differentially in particular kinds of reservoir hosts.

Dr. Randolph suggests that our experiments may have been confounded because Lyme disease spirochetes may have been inherited persistently within the laboratory colony of ticks used in our studies. Although an early observation points toward the possibility of such a mode of transovarial transmission (15), subsequent experimental evidence suggests that vertical transmission rarely, if ever, occurs (16). Inherited infection in nature would be exceedingly infrequent because spirochetes infect less than 1% of naturally questing larvae, both in North America and in Europe (17,18), and some of these larvae may have acquired infection by feeding partially on an infected host. We routinely seek evidence of spirochetal infection in each cohort of larval ticks used in our experiments but have never found spirochetes in a nonfed, laboratory-reared larva. Our reported frequency of experimental transmission of Lyme disease spirochetes from reservoir mice to vector ticks corresponds to that reported elsewhere (19-21).

Dr. Randolph's statistical analysis of our data confirms that the feeding success of nymphal ticks on robins exposed repeatedly to ticks varies nonsignificantly, supporting our conclusion that nymphal ticks readily feed repeatedly on tick-exposed robins. Although repeated nymphal infestations may protect inbred laboratory mice from tick-borne spirochetes (22), natural reservoir hosts, such as white-footed mice and American robins, remain susceptible to such spirochetes, regardless of prior exposure to ticks (1,23).
